# Suboptimal child spacing practice and its associated factors among women of child bearing age in Serbo town, JIMMA zone, Southwest Ethiopia

**DOI:** 10.1186/s40834-019-0085-1

**Published:** 2019-05-06

**Authors:** Girma Bacha Ayane, Kalkidan Wondwossen Desta, Birhanu Wondimeneh Demissie, Netsanet Abera Assefa, Emebet Berhane Woldemariam

**Affiliations:** 1 Department of Nursing, College of Medicine and Health Science, Jimma University, Jimma, Ethiopia; 2 School of Nursing and Midwifery, College of Health Science, Addis Ababa University, Addis Ababa, Ethiopia; 3 School of Nursing, College of Health Sciences and Medicine, Wolaita Sodo University, Sodo, Ethiopia

**Keywords:** Suboptimal, Birth interval, Women of child bearing age, Serbo town

## Abstract

**Introduction:**

Birth spacing is the time gaps between two consecutive life births. Optimal spacing until the next pregnancy is the resting period that allows the mother time to recover from pregnancy, and labor. Birth interval of 3 to 5 years increases maternal health and child survival and family planning programs have advocated this birth interval.

**Objectives:**

To assess prevalence of Suboptimal Child spacing practice and its associated factors among Women of Child bearing age in Serbo town, Jimma Zone Southwest Ethiopia.

**Methods:**

Community based cross sectional study was conducted on a total of 314 women of child bearing age from March to April 2017 who were selected by simple random sampling. A semi structured questionnaire which was pretested was used to collect the data. Data was checked for completeness and analyzed using SPSS V.20. Bi-variable logistic regression and multi- variable regression were done for predictor variables associated at *p*-value < 0.05 with the outcome variable.

**Result:**

The prevalence of short birth interval in this study was 59.9%. Independent predictors like age at first marriage (AOR: 2.10, 95% CI = 1.19, 3.69), sex of index child (AOR: 1.964, 95% CI = 1.05 3.96), educational status (AOR: 3.05,95% CI = 1.68, 3.83), duration of breastfeeding (AOR: 3.09, 95% CI = 1.38, 6.96) and use of modern contraceptives (AOR: 1.94, 95% CI = 1.09, 3.45) were found to be statistically associated with short birth interval.

**Conclusion and recommendation:**

Majority of the study respondents were practicing short birth interval. Education level, age at first marriage, having female child, short duration of breastfeeding and not using of modern contraceptives were factors associated with the outcome variable. Therefore awareness about modern contraceptive utilization, importance of breastfeeding as birth spacing mechanism and impact of early marriage are recommended.

## Background

Birth spacing is the time gaps between two consecutive life births also known as the inter-pregnancy interval [1]. Optimal spacing until the next pregnancy is the resting period between pregnancies that allows the mother time to recover from pregnancy to the next pregnancy. Longer time period between births allows the next pregnancy and birth to occur more likely to be at full gestation and family planning programs have advocated three and more years intervals between births for infant and child health and survival [2].

Three to five years birth interval is safer for both the mother and child. Globally, a birth interval of less than 18 months is associated with increased risk for Neonatal mortality (AOR =3.17), Infant mortality (AOR = 3.16), and Under-five mortality AOR = 2.81). Birth interval, less than 15 month is associated with increased risk for the mother like; third trimester bleeding (AOR = 1.7), Premature rupture of membranes (AOR = 1.7), Anemia (AOR = 1.3) and Puerperal endometritis (AOR = 1.3) [3, 4].

Short birth spacing has been continued to be a problem of both developed and developing countries like; Uganda and Zimbabwe, resulting in negative infant, child, and maternal health outcomes [5]. In USA, Pregnancies that occur with birth spacing less than 18 months are associated with delayed prenatal care and adverse birth outcomes, including preterm birth, neonatal morbidity, and low birth weight [6]. In Ethiopia also there is an increased risk of maternal health problems and death at a young age among those who give birth less than two years interval between the births [7].

Beyond the health implications, closely spaced birth intervals accelerate population growth and undermining development efforts. It makes difficult for women to become productive members of society, thereby limiting their contribution to economic development [8].

Determinant for the occurrence of short birth interval includes; maternal education, maternal age, early marriage, lack of optimal breastfeeding practice and inadequate knowledge, Attitude and practice towards modern contraceptives use and different socio-demographic factors [9, 10].

Even though interventions such as promoting female education and empowerment, awareness creation and enhancing breastfeeding practice and modern contraception utilization has been done, problem of birth spacing is still evident in most parts of African countries including Ethiopia. For instance, a previous study in southern Ethiopia showed that about more than half of (57.5%) women practicing shorter birth interval with the median birth interval length of 33 months [7, 11–15]. Optimum birth spacing has an invaluable benefit for the world in building capacity to promote healthy and economic independency through balancing and managing the growth of population and budget to ensure the production of fruitful generation. Identification of factors for suboptimal child spacing practice is critical for countries like Ethiopia which is the most populated next to Nigeria in Africa with estimate population size of 102,066,540 and with current fertility rate of 4.6. It will help to design interventional program and preventive strategies. Therefore, the aim of this study was to assess suboptimal child spacing practice and its associated factors among women of child bearing age in serbo town, Southwest Ethiopia.

## Methods

### Study design and setting

Community based cross - sectional study was conducted from March to April 2017 in Serbo Town Jimma Zone, Oromia Regional state, South - West Ethiopia. Serbo town is located in kersa district, Jimma Zone of Oromia regional state with an attitude of 1640 km above sea level. The town is located 345 km far from Addis Ababa, Capital city of Ethiopia and about 18 km from Jimma town. The study was conducted in two kebeles (smallest administrative division in Ethiopia), namely Omaticha kebele and Wayu kebele. The town has two kebeles with total populations of 7450, of this 3650 were females. There were a total of 1649 Child bearing age mothers in the town.

### Study population

The source populations were all women’s of child bearing age groups (15–49) in Serbo town. And all randomly selected women of child bearing age group in Serbo town were the study population.

## Eligibility criteria

### Inclusion criteria

All women of child bearing age group (15–49) having at least two children were included in the study.

### Sample size determination

Sample size was calculated by using the formula for single population proportion and by considering, 35.8% proportion of optimal birth interval in Lemo District, Southern Ethiopia [13] 95% level of confidence, 5% margin of error and 10% none response rate, the sample size was 388. Since the total population of reproductive age mothers in Serbo town is less than 10,000 correction formula was used to get the final sample size of 314 women’s.

### Sampling techniques

The sample was taken from two kebele’s (smallest administrative division in Ethiopia) in Serbo town, namely Omoticha kebele and Wayu Kebele and the study was conducted in these two kebele’s. The calculated sample size was allocated proportionally to the size of populations in each kebeles. Household was selected with an interval of K for each kebele, where K was the total number of household in each kebeles divided by the total number of study subjects in each kebele’s which was found to be **5** (K = 5, every 5th household**)** for both kebele’s and the first house hold to be started from was selected by lottery method.

### Method of data collection

Data was collected using a semi-structured questionnaire which was adopted and modified from different literature [13]. The questionnaire contains: socio-demographic characteristic of the study respondents, awareness of the study respondents on birth spacing, birth history of the study respondent, practice of breastfeeding and use of modern contraceptives. All eligible mothers of child bearing age were interviewed by using semi-structured questioners. The data was collected by four diploma holder nurses and two BSc. holder Environmental health professionals and the data collection was supervised by two MSc. holder nurses.

### Data quality and control

The questionnaire were prepared in English version and translated into local languages (Amharic and Afan Oromo) and then back translated into English to maintain its consistency. Three days orientation training was given on the process of data collection for data collectors and supervisors. A pretest was done by 5 % of the study population three weeks before the actual data collection to evaluate the clarity of questions and validity of the instrument and reaction of respondents to the questions.

### Study variables

### Dependent variable

- Suboptimal birth spacing

### Independent variables

Socio-demographic variables (Age, marital status, educational level, religion, occupation), fertility history, contraceptive utilization, inconsistent breastfeeding.

### Operational definitions

#### Optimal birth interval

it denotes to 3–5 years’ birth interval (including 3 and 5 years) between the birth of the child under study and the immediately preceding live and surviving birth to the mother.

#### Suboptimal birth interval

it refers to less than 3 years’ birth interval between the birth of the child under study and the immediately preceding live and surviving birth to the mother.

#### Long birth interval

it refers greater than 5 years’ birth interval between the birth of the child under study and the immediately preceding live and surviving birth to the mother.

### Data entry and analysis

The data was cleaned manually, coded and entered into Epi data version 3.1 and exported to SPSS version 22.0 software for further analysis. Bi-variable logistic regression analysis was used to see significance of association between dependent and each independent variable. Candidate variables for the final model (multivariate binary logistic regressions) were identified at *p* - value < 0.20. Variables which had significant association with the outcome variable during bi-variable logistic regression analysis was introduced in to multivariable binary logistic regression and finally, predictors of suboptimal child spacing were identified at p – value < 0.05, 95% CI.

## Results

### Socio - demographic characteristics of mothers

A total of 314 mothers participated in the study, with response rate of 100%. From the total respondents one hundred and nine (34.7%) were in the age group of 25–29 with mean age of 30.84 (SD ± 5.956) years. Most, 292 (93.0%) of the respondents were married. Majority of the respondents 215 (68.5%) age at first marriage was 18 years and above with mean of 18.38 (SD ± 2.41) years of age. Two hundred and thirty five (74.8%) were Muslim religious followers. Almost one third of 115(36.6%) respondents were illiterate and majority of them 209 (66.6%) were housewives. More than half, 171 (54.5%) of the study participants had monthly income of less than 1000 Ethiopian birr (37 US Dollar) **(**Table 1).

**Table 1:** Socio-demographic characteristic of mothers of child bearing age in Serbo town Jimma Zone Southwest Ethiopia, 2017 (*N* = 314)

Variables	Category	Frequency	Percent
Age of the mother	15–19	2	.6
	20–24	32	10.2
	25–29	109	34.7
	30–34	78	24.8
	35–39	59	18.8
	> = 40	34	10.8
Marital status	Married	292	93.0
	Others *	22	7.0
Age at first marriage	< 18 years	99	31.5
	> = 18 years	215	68.5
Religion	Orthodox	34	10.8
	Protestant	34	10.8
	Muslim	235	74.8
	Others**	11	3.5
Ethnicity	Oromo	266	84.7
	Amhara	18	5.7
	Gurhage	17	5.4
	Tigray	6	1.9
	Others **^a^	7	2.2
Education	Illiterate	115	36.6
	Read and write	37	11.8
	Elementary	111	35.4
	Secondary and above	51	16.2
Occupation	Employ of government or NGO	43	13.7
	House wife	209	66.6
	Merchant	41	13.1
	Others **^b^	21	6.7
Monthly income	< 1000 birr (37 US dollar)	171	54.5
	1000–1999 birr (37–74 $)	91	29.0
	2000–2999 birr (74–111$)	37	11.8
	≥ 3000 birr (≥112$)	15	4.8

*others: single, Divorced & widowed. **others: Wakefata & Catholic. **^a^ Others: Wolayita, Silte & Dawuro. **^b^others: Farmer, Student and daily workers

### Birth history, awareness about birth spacing and breastfeeding practice

Most of the respondents 286 (91.1%) were informed about modern contraceptives methods used by male and female to space and limit child birth. Majority 265 (84.4%) of the study respondents agreed that an optimal birth interval has health advantages both for the mother and child. Of the total respondents 47 (15.0%) did not know the health disadvantages of short birth interval for the mother and the child. Concerning with the preference of birth interval among mothers; more than half, 163 (51.9%) prefer an optimal birth interval. Almost half of the respondents 159 (50.6%) have three to four children and about thirty eight (12.1%) mothers reported child death soon after birth. Two hundred and eighty three (90.1%) of mothers have breastfed their last child, but about 31(9.9%) of them have not yet breastfed their last child due to different reasons. Among those who breastfed their last child, above three forth, 217(76.4%) breastfed their last child for 24 months and above whereas18 (6.3%) for less than 24 months (Table 2).

**Table 2:** Birth history, knowledge about birth spacing and breastfeeding practice among mothers of child bearing age in Serbo town Jimma Zone, Southwest Ethiopia, 2017 (*N* = 314)

Variables	Category	Frequency	Percent
Heard about modern contraceptives during two consecutive births	Yes	286	91.1%
	No	28	8.9%
Optimal birth interval has health advantage for the mother and the child	Yes	265	84.4%
	No	39	12.4%
	don’t know	10	3.2%
Short birth interval has health disadvantages for the mother and the child	Yes	260	82.8%
	No	47	15.0%
	don’t know	7	2.2%
Number of children born alive	2	110	35.0%
	3–4	159	50.6%
	≥5	45	14.3%
Child died soon after birth	Yes	38	12.1%
	No	276	87.9%
Mother’s need to have more children after last pregnancy	Yes	191	60.8%
	No	123	39.2%
Time to become pregnant after last child	Soon after	47	24.6%
	Wait until later	144	75.4%
Mother’s preference of birth interval (BI) in years	< 36 months	113	36.0%
	36–60 months	163	51.9%
	≥60 months	25	8.0%
	I don’t know	13	4.1%
Sex of the last child	Male	110	35.0%
	Female	204	65.0%
Breastfeed the last child	Yes	283	90.1%
	never breastfeed	31	9.9%
Duration of breastfeeding	0–11 months	19	6.7%
	12–23 months	49	17.3%
	> = 24 months	217	76.0%
Reasons for never breastfed	new pregnancy	5	16.1%
	mother was sick	14	45.2%
	Other^a^	12	38.7%
Mother’s agreement on when to stop breastfeeding	< 24 months	57	17.9%
	≥24 months	257	82.1%

^a^others include, mothers don’t have breast milk b/c of unknown reason, the child refused to suck

### Distribution of birth intervals practice

More than half 188(59.9%) of the mothers were practicing short birth interval less than 36 months, followed by 112 (35.7%), and 14(4.5%) practicing optimal and long birth interval between their last two children respectively.

### Awareness and utilization of modern contraceptive

Two hundred and sixty six (84.7%) of the study respondents had information about modern contraceptives used to space and limit birth. Nearly two third (62.7%) of the study respondents had used those methods before the last child, 162(82.2%) for spacing purpose. One hundred fifty three (89.5%) of them were getting the service from health center and fifty one (35.7%) of those who did not use modern contraceptive mentioned religion as the main reason for not using (Table 3).

**Table 3:** Awareness and utilization of modern contraceptive among mothers of child bear age in Serbo town Jimma Zone, Southwest Ethiopia, 2017(*N* = 314)

Variables		Category	Frequency	Percent
mothers have an awareness about modern Contraceptive		Yes	266	84.7%
		No	48	15.3%
Mother used modern contraceptives before the last child		Yes	197	62.7%
		No	117	37.3%
Purpose of using modern contraceptives		birth spacing	162	82.2%
		birth limiting	35	17.8%
Current use of modern contraceptives by mother		Yes	171	54.5%
		No	143	45.5%
The place where mother gets modern contraceptives		health post	14	8.2%
		health center	153	89.5%
		Hospital	25	14.6%
		private sector	5	2.9%
Reason of the mother not to use modern contraceptives		desire to have more children	27	18.9%
		health problem	15	10.5%
		religious reason	51	35.7%
		husband not willing	20	14.0%
		Other^a^	30	21.0%

^a^Others include, lack of service, lack of information, no husband, naturally adjusted spacing, lack of interest of modern contraceptives

### Birth spacing methods utilization

**Fig. 1:**
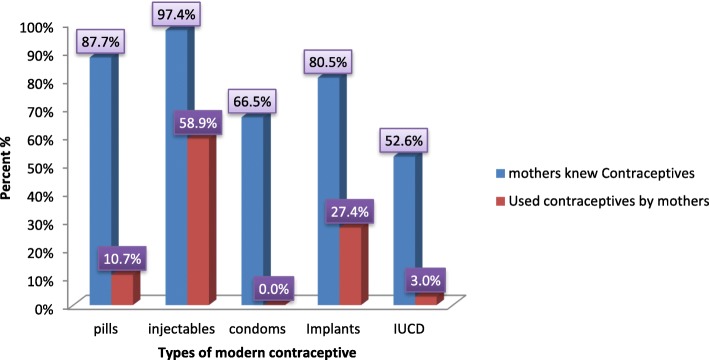
Awareness and modern contraceptives utilization among mothers of child bearing age in Serbo town Jimma Zone, Southwest Ethiopia, 2017

Even though more than half of the study respondents knew different modern contraceptives used to space and limit child birth, few of them used some of those contraceptives. This indicates that the awareness level about modern contraceptives of the study respondents was high while their actual utilization or practice of the methods was too low. The most frequently known contraceptive was injectable 259 (97.4%) followed by pills 233 (87.7%). Injectable was also the most frequently used 116 (58.9%) and more than half, 177 (66.5%) of the study respondents knew about condoms, but none of them utilized (Fig. 1).

### Predictors of short birth interval

In bi-variable logistic regression, Educational status, age at first marriage, sex of the index child, death of child soon after birth, mother’s preference of birth interval, breastfeeding of previous to last child, duration of breast feeding and use of modern contraceptives were the variables that showed statistically significant association with the outcome variable. Those variables that had association in bi variable logistic regression were introduced into multiple logistic regressions.

The likelihood of practicing short birth interval was about 2 times more likely in mothers who were married at age less than 18 years as compared to mothers who were married at age 18 years and above (AOR = 2.10, 95% CI = 1.19, 3.69). The odds of practicing short birth interval was about 3 times more likely in respondents who had no formal education as compared to those who had formal education (AOR: 3.09,95% CI = 1.68, 3.83). In the same way, the probability of having female index child was about 2 times more likely to increase the chance of getting short birth as compared to those mothers who had male index child (AOR = 1.96,95% CI = 1.046, 3.96). Study respondents who preferred birth spacing of less than 36 months were about 4 times more likely to have short birth interval as compared to those who preferred 36–60 months (AOR = 4.71,95% CI = 2.46,7.53). Mothers who had a duration of breast feeding < 24 months were 3 times more likely to have short birth interval as compared to those who breastfeed for ≥24 months (AOR = 3.09, 95% CI 1.38, 6.96). The odds of not using modern contraceptives in the mothers who practiced a short birth interval were 2 times higher than among those using modern contraceptives (AOR; 1.93, 95% CI = 1.09, 3.45) **(**Table 4**).**

**Table 4:** Factors associated with short birth interval among mothers of child bearing age in Serbo town Jimma Zone, Southwest Ethiopia, 2017 (*N* = 314)

Variables	category	Short Birth Interval		COR(95%CI)	AOR(95%CI)
		Yes	No		
		*N* (%)	N (%)		
Age at first marriage	< 18 years	72 (22.9%	27 (8.6%)	2.28 (1.36,3.82)	2.09 (1.19,3.69)*
	≥18 years	116 (36.9%)	99 (31.5%)	1.00	1.00
Sex of index child	Male	33 (10.5%)	30 (9.6%)	1.00	1.00
	Female	93 (29.6%)	158 (50.6%)	1.87 (1.07,3.26)	1.96 (1.05,3.96)*
Death of child soon after birth	Yes	29 (9.2%)	9 (2.9%)	1.00	
	No	159 (50.6%)	117 (37.3%)	2.371 (1.08,5.19)	
Preference of birth interval	< 36 months	73 (23.2%)	90 (28.7%)	5.733 (3.23,10.17)	4.71 (2.46,7.53)*
	≥60 months	15 (4.8%)	10 (3.2%)	1.85 (0.78,4.36)	0.26 (0.08,0.90)
	don’t know	7 (2.2%)	6 (1.9%)	1.44 (1.46,4.47)	0.27 (0.077,0.920)
	36–60 months	93 (29.6%)	20 (6.4%)	1.00	1.00
Educational status	No formal education	136 (43.3%)	92 (29.3%)	3.09 (1.68,3.71)	3.05 (1.60,3.83)*
	Formal educated	52 (16.6%)	34 (10.8%)	1.00	1.00
Breastfeeding of previous to last child	Yes	120 (38.2%)	163 (51.9%)	1.00	
	No	6 (2%)	25 (8.0%)	3.07 (1.22,7.71)	
Duration of breast feeding	< 24 months	47 (15.0%)	10 (3.2%)	3.84 (1.83,7.83)	3.09 (1.38,6.96)*
	≥24 months	141 (44.9%)	116 (36.9%)	1.00	1.00
Use of modern contraceptives	No	86 (27.4%)	31 (9.9%)	2.58 (1.57,4.25)	1.94 (1.09,3.45)*
	Yes	102 (32.5%)	95 (30.3%)	1.00	1.00

*Significant at *p*-value ≤0.05

## Discussion

The prevalence of short birth interval in this study was found to be 59.9%. The remaining, 35.7 and 4.5% were practicing optimal and long birth interval respectively. Although, 36–60 months of interval between births is the currently recommended, still the prevalence of short birth interval outweighs the optimal one. This finding is almost consistent with a study conducted in southern Ethiopia, 57.6% of mothers were practicing short birth interval and 35.8% were practicing optimal birth interval and the remaining were long birth interval. This might be due to relative similarity in socio-economic background of the study respondents. Again, this finding was relatively similar with the study conducted in Mozambique in which 46, 35 and 19% of the women were practicing short, optimal and long birth interval respectively. However, in the current study, long birth interval was lower than study conducted in Mozambique [10, 15]. The discrepancy might be due to difference in demographic background of the study respondents of the two countries.

Those women who had married at age less than 18 years were about 2 times more likely to practice short birth interval as compared to those who had married at age 18 years or above. This finding was consistent with the study done in Uganda, Zimbabwe and Jordan [16, 17].

Sex of the index child was associated with short birth interval. According to the result of this study women’s who had female index child were about 3 times more likely to have short birth interval as compared to mothers who had male index child which was consistent with studies done in Arba - minch Zuria District and Southern Ethiopia [7, 8].

Mothers who had no formal education were about 3 times more likely to have short birth interval. A similarly study conducted in Arba-Minch showed the same finding with the current study (AOR = 3.40, 95% CI: 1.80, 6.43) [7, 8]. This is also consistent with a study conducted in Illubabor zone, South West Ethiopia (AOR = 2.56, 95% CI: 1.60, 3.42) [18]. This might be due to knowledge gap about family planning methods, where to get, how to use and inability to read different advertisements about family planning.

The other predictor of short birth interval was duration of breastfeeding. Mothers who breastfeed their child for less than 24 months were about 3 times more likely to practice short birth interval as compared to those who breastfeed ≥24 months which is in line with a study conducted in Illubabor zone, South West Ethiopia (AOR = 5.36, 95% CI: 3.43, 6.34) [18]. This was lower than a study conducted in Southern Ethiopia which revealed that breastfeeding for less than 24 months was 30.8 times more likely to practice short birth interval [8]. This is due to lactation amenorrhea and this variation might be due to the difference in duration of breastfeeding practice.

The odds of not using modern contraceptive was about 2 times to have short birth interval as compared to those users of modern contraceptives (AOR = 1.94, 95% CI 1.09, 3.45). Which was similar with a study conducted in Arba-Minch district, Lemo district and rural communities of southern Ethiopia [7, 8, 15]. This is lower than a study conducted in Illubabor zone, South West Ethiopia in which those who are not using contraceptive were 4.12 times more likely to have short birth interval (AOR = 4.12, 95% CI: 2.7, 5.82) [18]. This variation might be due to the difference in health access for family planning service, unmet need due to distance and variation in awareness of women’s on family planning methods.

## Conclusions

The prevalence of short birth spacing among mothers of childbearing age in serbo town was high (59.9%) which shows mothers were giving birth with short birth interval.

Early marriage, sex of the index child, mother’s preference of birth interval, educational status, and short duration of breast feeding and lack of modern contraceptive utilization were independent predictors of short birth interval. Therefore Minister of health and Regional Health bureau should strengthen actions to prevent early marriage and increase mothers’ awareness of modern contraceptive and importance of exclusive breastfeeding for the first six month and continue breast feeding until two years old through health extension professionals, and attention should be given on actions to increase modern contraceptive utilization.

## Abbreviations

AOR: Adjusted odds ratio
CI: confidence interval
SD: Standard deviation
SPSS: Statistical Package for Social Sciences.
